# Supplementary material for: Galactooligosaccharides Promote Gut Barrier Integrity and Exert Anti-Inflammatory Effects in DSS-Induced Colitis Through Microbiota Modulation
Source: Int J Mol Sci. 2025 Aug 18;26(16):7968. doi: 10.3390/ijms26167968 (PMC12386736; doi:10.3390/ijms26167968)
Supplement: Supplementary file 1 [file ijms-26-07968-s001.zip › ijms-3768182-supplementary.pdf]

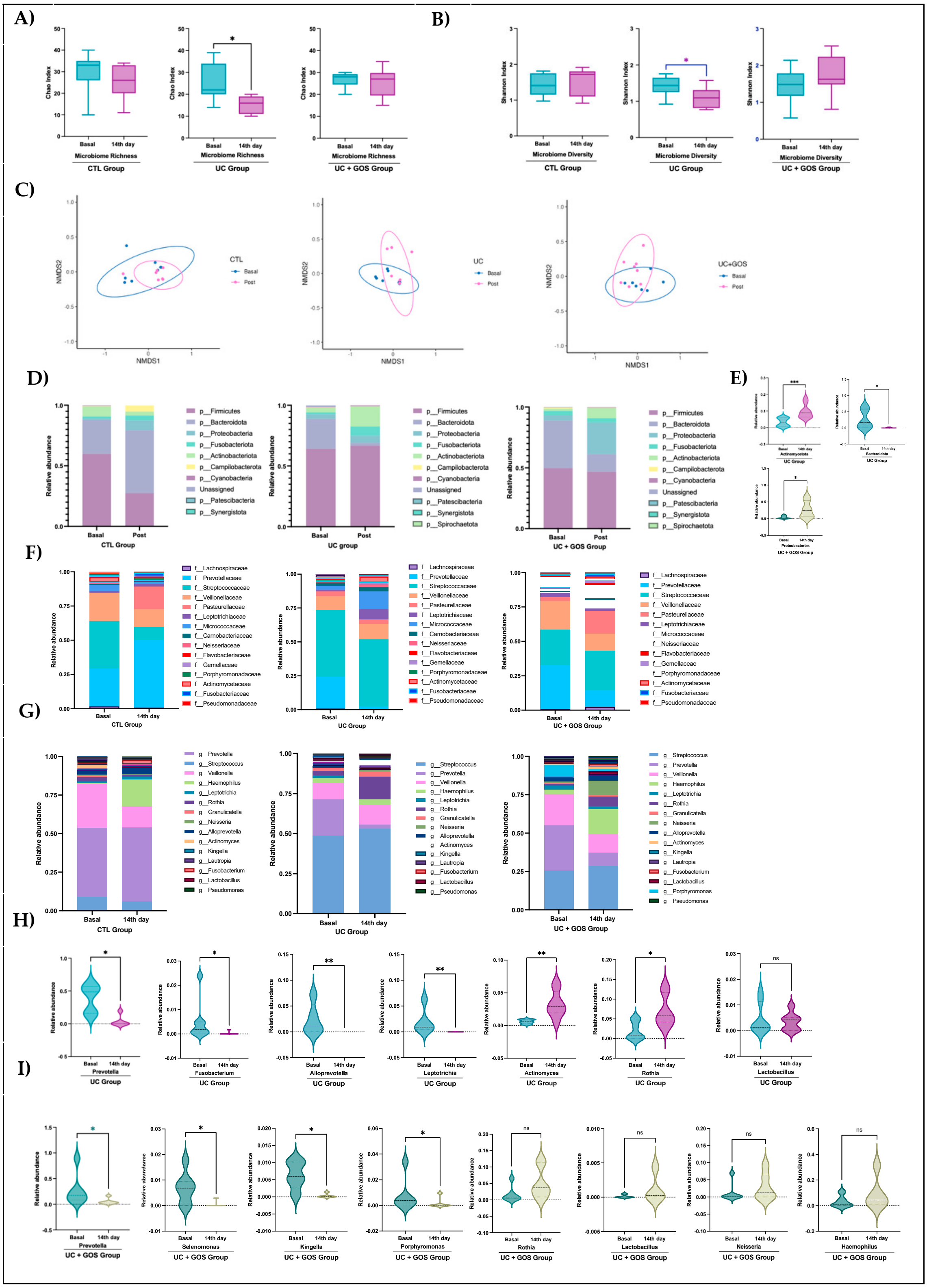
A)
B)
C)
D)
E)
F)
G)
H)
I)
Chao Index
Basal
14th day
Microbiome Richness
CTL Group
UC Group
UC + GOS Group
Shannon Index
Microbiome Diversity
NMDS1
NMDS2
CTL
UC
UC+GOS
Post
Relative abundance
p__Firmicutes
p__Bacteroidota
p__Proteobacteria
p__Fusobacteriota
p__Actinobacteriota
p__Campilobacterota
p__Cyanobacteria
Unassigned
p__Patescibacteria
p__Synergistota
p__Spirochaetota
UC group
Actinomycetota
Bacteroidota
Proteobacteria
f__Lachnospiraceae
f__Prevotellaceae
f__Streptococcaceae
f__Veillonellaceae
f__Pasteurellaceae
f__Leptotrichiaceae
f__Micrococcaceae
f__Carnobacteriaceae
f__Neisseriaceae
f__Flavobacteriaceae
f__Gemellaceae
f__Porphyromonadaceae
f__Actinomycetaceae
f__Fusobacteriaceae
f__Pseudomonadaceae
g__Prevotella
g__Streptococcus
g__Veillonella
g__Haemophilus
g__Leptotrichia
g__Rothia
g__Granulicatella
g__Neisseria
g__Alloprevotella
g__Actinomyces
g__Kingella
g__Lautropia
g__Fusobacterium
g__Lactobacillus
g__Porphyromonas
g__Pseudomonas
Prevotella
Fusobacterium
Alloprevotella
Leptotrichia
Actinomyces
Rothia
Lactobacillus
Selenomonas
Kingella
Porphyromonas
Neisseria
Haemophilus
ns

Figure S1. Gut microbiota composition pre-and post-colitis induction and GOS treatment. A) Box plot representing the minimum, median, and maximum values of microbiota richness of CTL, UC and UC + GOS groups. **B)** Box plot representing the minimum, median, and maximum values of microbiota diversity of CTL, UC and UC + GOS groups. **C)** Non-metric multidimensional scaling (NMDS) analysis of gut microbiota composition of each group comparing the pre- and post- state**. D)** Relative abundance of the main bacterial phyla in each group in pre- and post- states. **E)** Violin plots showing significant changes at the phylum level in UC and UC + GOS groups. **F)** Comparison of the relative abundance of key bacterial families among groups in pre- and post- states. **G)** Comparison of the relative abundance of gut microbiota genera among groups. **H)** Violin plots illustrating the relative abundance of gut microbiota genera with significant differences between the pre- and post- states in UC group**. I)** Violin plots illustrating the relative abundance of gut microbiota genera with significant differences between the pre- and post- states in UC + GOS group. *$p \leq 0.05$, **$p \leq 0.005$, ***$p \leq 0.0001$. Data are presented as median with 95% confidence intervals. Violin plots display median and quartiles lines, along with the data distribution. A total of 7-8 mice per group were used for gut microbiota analysis.

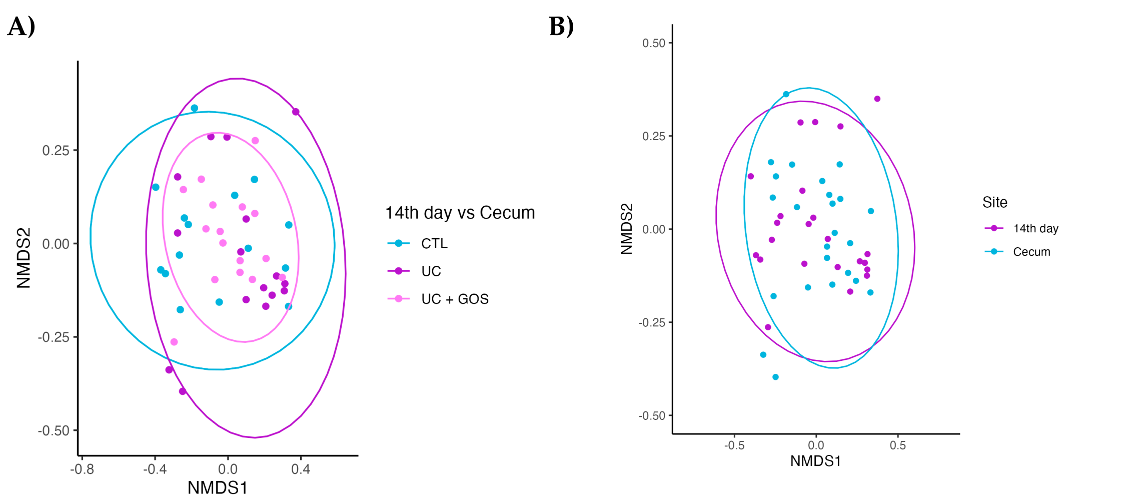

Figure S2. NMDS analysis of gut microbiota composition between 14-day and cecum feces. **A)** Non-metric multidimensional scaling (NMDS) analysis of gut microbiota composition across experimental groups. **B)** NMDS analysis of gut microbiota composition based on sample site (day 14 vs. cecum feces). A total of 7-8 mice per group were analyzed for gut microbiota composition.

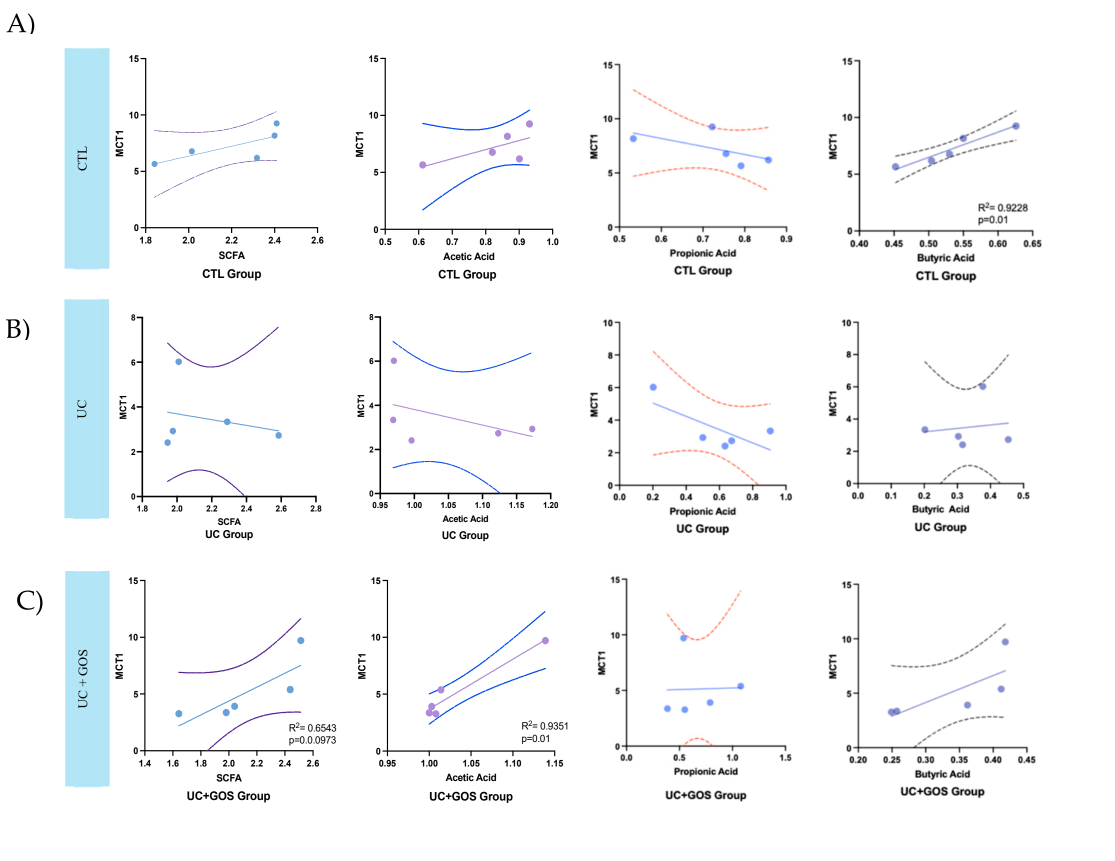

Figure S3. Correlation analysis between MCT1 expression and SCFAs concentrations in cecum feces. A) Regression plots showing the relationship between MCT1 expression and total SCFA concentration, as well as individual correlation for acetic, propionic and butyric acids in mice bellowing to CTL group. **B)** Regression plots showing the relationship between MCT1 expression and total SCFA concentration, as well as individual correlation for acetic, propionic and butyric acids in mice bellowing to UC group. **C)** Regression plots showing the relationship between MCT1 expression and total SCFA concentration, as well as individual correlation for acetic, propionic and butyric acids in mice bellowing to UC + GOS group. All correlations were analyzed using Pearson's correlation. The figure includes linear regression line with 95% confidence bands.

Table S1. Primers sequences and annealing temperature used for gene expression analysis.			
Primer	Sequence 5′-3′	Annealing temperature °C	Reference
MCT1	F: CATTGGTGTTATTGGAGGTC<br>R: GAAAGCCTGATTAAGTGGAG	55	[89]
MCT4	F: TCAATCATGGTGCTGGGACT<br>R: TGTCAGGTCAGTGAAGCCAT	57	[89)
ZO-1	F: ACTCCCACTTCCCCAAAAAC<br>R: CCACAGCTGAAGGACTCACA	60	[88]
JAM-A	F: CTGATCTTTGACCCCGTGAC<br>R: ACCAGACGCCAAAAATCAAG	60	[88]
Occludin	F: CGGTACAGCAGCAATGGTAA<br>R: CTC CCC ACC TGT CGT GTA GT	56	[94]
Claudin-1	F: AGGTCTGGCGACATTAGTGG<br>R: TGGTGTTGGGTA AGAGGTTG	58	[93]
Claudin-2	F: TGCGACACACAGCACAGGCATCAC<br>R: TCAGGAACCAGCGGCGAGTAGAA	60	[90]
Claudin-4	F: TCGTGGGTGCTCTGGGGATGCTT<br>R: GCGGATGACGTTGTGAGCGGTC	60	[90]
IL-6	F: AGAGACTTCCATCCAGTTGC<br>R: TCCTTAGCCACTCCTTCTGT	60	[91]
IL-17	F: ATCCCTCAAAGCTCAGCGTGTC<br>R: GGGTCTTCATTGCGGTGGAGAG	60	[92]
IL-10	F: TAGAGCTGCGGACTGCCTTC<br>R: CTTCACCTGCTCCACTGCCT	60	[91]
TNF-a	F: CTGTGAAGGGAATGGGTGTT<br>R: GGTCACTGTCCCAGCATCTT	62	[91]
Beta-Actin	F: CCATCATGAAGTGTGACGTGG<br>R: GTCCGCCTAGAAGCATTTGCG	62	[87]
